# Research and Remedies

**Published:** 1916-06-24

**Authors:** 


### RESEARCH AND REMEDIES.

### The "Innocence" of Gall-Stones.

Ten years ago we heard a great deal about "innocent" gall-stones, which meant that gall-stones existed without symptoms, and that their presence was not suspected until *post-mortem* examination brought them to light. We cannot now escape the conviction that the gall-stones did cause symptoms, and that we, as diagnosticians, and not the gall-stones, were "innocent."—*Wm. Mayo.*

### Syphilis and Chorea.

Dr. Henry Koplik concludes that all chorea of the Sydenham type is of an infectious nature, though, in spite of blood cultivation in many cases, he has never been able to isolate any micro-organism. He does not accept the suggestion that syphilis plays any part in the etiology of the disease. In ten cases he tested the blood for the Wassermann reaction, and in no instance was a positive result obtained. Further, he insists that children the subjects of hereditary syphilis are not affected with chorea in any larger proportion than those free from specific taint. Again, after administering neo-salvarsan to nine cases, he did not find that the course of the disease was modified. In one of these patients the use of the drug was followed by nephritis. This latter result, it may be noted, has been observed in other instances, and an elaborate study of the subject has been made by Dr. Wade H. Brown. Dr. Koplik's results do not confirm the claim that has been made by some French observers who have used salvarsan in the treatment of chorea.—*Archives of Pediatrics.*

